# Hyperinflation and reduced diffusing capacity predict prognosis in SCLC: value of extended pre-therapeutic lung function testing

**DOI:** 10.1177/17534666231199670

**Published:** 2023-11-24

**Authors:** Kathrin Kahnert, Lilli Maria Lempert, Jürgen Behr, Laura Elsner, Toki Bolt, Amanda Tufman, Diego Kauffmann-Guerrero

**Affiliations:** Department of Medicine V, University Hospital, LMU Munich, Munich, Germany; Comprehensive Pneumology Center Munich (CPC-M), Member of the German Center for Lung Research (DZL), Munich, Germany; Department of Medicine V, University Hospital, LMU Munich, Munich, Germany; Department of Medicine V, University Hospital, LMU Munich, Munich, Germany; Comprehensive Pneumology Center Munich (CPC-M), Member of the German Center for Lung Research (DZL), Munich, Germany; Department of Medicine V, University Hospital, LMU Munich, Munich, Germany; Comprehensive Pneumology Center Munich (CPC-M), Member of the German Center for Lung Research (DZL), Munich, Germany; Department of Medicine V, University Hospital, LMU Munich, Munich, Germany; Comprehensive Pneumology Center Munich (CPC-M), Member of the German Center for Lung Research (DZL), Munich, Germany; Department of Medicine V, University Hospital, LMU Munich, Munich, Germany; Comprehensive Pneumology Center Munich (CPC-M), Member of the German Center for Lung Research (DZL), Munich, Germany; Department of Internal Medicine V (Pneumology/Thoracic Oncology), University Hospital, LMU Munich, Ziemssenstraße 1, Munich 80336, Germany; Comprehensive Pneumology Center Munich (CPC-M), Member of the German Center for Lung Research (DZL), Munich, Germany

**Keywords:** body plethysmography, diffusion capacity, hyperinflation, lung function, SCLC

## Abstract

**Background:**

Small cell lung cancer (SCLC) is characterized by aggressive growth and poor prognosis. Although SCLC affects nearly exclusively heavy smokers and leads to frequent respiratory symptoms, the impact of pre-therapeutic lung function testing in SCLC is sparely investigated until now. Therefore, we sought to examine whether we could find prognostic markers in pre-therapeutic lung function testing of SCLC patients.

**Patients and Methods:**

We retrospectively analysed a cohort of 205 patients with the diagnosis of SCLC between 2010 and 2018. Pre-therapeutic values of spirometry, body plethysmography and measurement of diffusing capacity was extracted from patients’ charts. Comparisons between groups were performed using the Mann–Whitney *U-*test or by chi-square tests as appropriate. Kaplan–Meier analyses and COX-regression models were performed to correlate lung function parameters with patients’ outcome.

**Results:**

Airway obstruction itself, or the diagnosis chronic obstructive pulmonary disease (COPD) based on GOLD definitions did not correlate with survival in SCLC patients. Hyperinflation measured by increased residual volume and residual volume to total lung capacity ratio (log-rank *p* < 0.001) and reduced diffusing capacity (log-rank *p* = 0.007) were associated with reduced survival. Furthermore, patients with hyperinflation as well as impairments in gas exchange representing an emphysematic phenotype had the worst outcome (log-rank *p* < 0.001).

**Conclusion:**

We recommend including body plethysmography and measurement of diffusing capacity in the pre-therapeutic assessment of SCLC patients. Our findings suggest that reduction of hyperinflation may lead to better outcome in SCLC patients. Thus, in addition to effective tumour therapy, adequate therapy of the comorbidity of COPD should also be provided. In particular, measures to reduce hyperinflation by means of dual bronchodilation as well as respiratory physiotherapy should be further assessed in this setting.

## Introduction

Small cell lung cancer (SCLC) accounts for about 15% of all diagnosed lung cancers and is characterized by rapid and aggressive growth.^1^ Despite good initial response to first-line chemotherapy, median survival of advanced SCLC patients remains poor, ranging from 7 to 10 months. The 1-year overall survival (OS) is only about 20–40%.^2^ However, prognosis heavily varies between different patients and reliable prognostic markers are needed. Several prognostic factors have been identified in the last decades including high levels of lactate dehydrogenase (LDH)^3,4^ and inflammatory markers.^5–7^ The major risk factor for SCLC development is smoking,^2^ which is on the other hand also a risk factor for the development of chronic obstructive pulmonary disease (COPD). Therefore, COPD seems to be clearly linked to the development of SCLC,^8^ but until now there is no strong evidence that this comorbidity affects prognosis in SCLC patients.

Spirometry is widely used to evaluate patient’s fitness before lung cancer treatment, and is of particular relevance before surgery, irradiation or systemic treatments, that is, chemotherapy and immunotherapy. A few small studies demonstrated a correlation of spirometric measurements including forced expiratory volume in 1 s (FEV_1_), forced vital capacity (FVC) or their ratio FEV_1_/FVC to the outcome in lung cancer patients.^9,10^ Some authors found a prognostic impact of impaired FEV_1_ and the presence of COPD in non-small cell lung cancer (NSCLC), but not in SCLC.^11–13^ However, all previous studies focused on spirometric lung functions parameters.

To the best of our knowledge, there are no data on the value of extended pre-therapeutic lung function testing including body plethysmography and measurement of diffusing capacity in SCLC patients. Thus, this study aims at investigating the prognostic value of pre-therapeutic extended lung function testing in patients with SCLC.

## Patients and methods

### Study population

A total of 205 patients diagnosed and treated with SCLC between 2010 and 2018 at our tertiary care lung cancer centre were included into this retrospective analysis. Patient characteristics in terms of gender, age, ethnicity, performance status, tumour stage and smoking status were collected from patient charts (paper-based and electronic records). Age was calculated at the time of diagnosis. Treatment response was evaluated by CT- or positron emission tomography (PET-CT)-based monitoring using the Response Evaluation Criteria in Solid Tumors 1.1 (RECIST).

### Lung function values

Pre-therapeutic lung function values (spirometry, body plethysmography, diffusing capacity) were collected from patient chats and comprised the following values: FEV_1_, FVC and their ratio FEV_1_/FVC, peak expiratory flow (PEF), total lung capacity (TLC), residual volume (RV), specific resistance (sRtot) and diffusing capacity of the lungs for carbon monoxide (DLCO). Predicted values for spirometry^14,15^ and diffusing capacity^16^ were taken from the Global Lung Function Initiative, for body plethysmography from the European Coal and Steel Community (ECSC).^17^

### Statistical analyses

For data description, mean values and standard deviations were used. Comparisons between groups were performed by the Mann–Whitney *U-*test, or by chi-square-tests in case of categorical variables. Estimation of PFS and OS was carried out using the Kaplan–Meier method with the log-rank test. For multivariate survival analysis, COX-regression models were used including variables with significant results in the univariate analysis. To define optimal cut-off values, we used a Receiver operating characteristic (ROC) analyses with the Youden criterion using the overall survival grater or smaller 2 years as dependent variable. The level of statistical significance was determined at *p* < 0.05. All statistical analyses were performed using SPSS 25 statistical software (IBM Corp., Armonk, NY, USA).

### Data analysis plan

**Figure 1.**
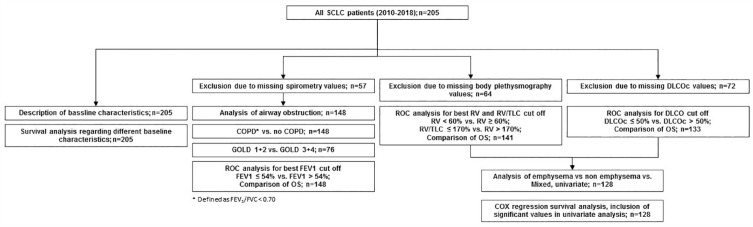
Data analysis Flow-Chart.

Figure 1 provides a data analysis flow-chart indicating the performed analyses and the numbers of included patients.

## Results

### Representative SCLC patient cohort

The total study population comprised *n* = 205 patients with a higher percentage of male patients (53.2%). The mean age at diagnosis was 65.3 years. Most patients were former, or current smokers (72.7%) and were diagnosed in locally advanced or metastasized stage (90.1%). Patients’ baseline characteristics are summarized in Table 1.

**Table 1.** Baseline characteristics of the study cohort.

Variable	*N* = 205
Gender	
Male	109 (53.2)
Female	96 (46.8)
Age	
Total	65.3 ± 9.5
Male	65.8 ± 9.8
Female	64.8 ± 9.1
Ethnics	
Caucasian	204 (99.5)
Asian	1 (0.5)
Initial ECOG	
0	34 (16.6)
1	72 (35.1)
2	32 (15.6)
3	11 (5.4)
4	4 (2.0)
Unknown	52 (25.4)
Smoking status	
Former smoker	67 (32.7)
Current smoker	82 (40)
Never smoker	4 (2.0)
Unknown	52 (25.4)
COPD status	
No COPD	72 (35.1)
GOLD 1	6 (2.9)
GOLD 2	43 (21.0)
GOLD 3	24 (11.7)
GOLD 4	3 (1.5)
Not available	57 (27.8)
UICC stage	
I	1 (0.5)
II	15 (7.3)
III	49 (23.9)
IV	136 (66.3)
Unknown	4 (2.0)
Tumour localization	
Right	101 (49.3)
Left	90 (43.9)
Not defined	14 (6.8)

**Figure 2.**
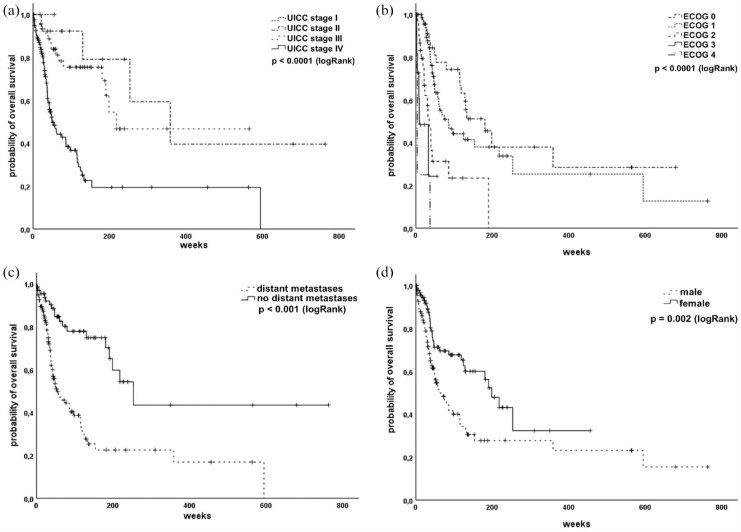
Baseline characteristics defining the prognosis in our SCLC cohort. (a) Patients with higher UICC stage show reduced prognosis. (b) Patients’ performance status predicts survival. (c) Patients with distant metastases at time of diagnosis show reduced overall survival. (d) Woman with SCLC seem to have a better outcome than male patients. SCLC, small cell lung cancer.

We tested several baseline characteristics regarding their impact on OS. As expectable, higher tumour stage, reduced performance status and existence of distant metastases were significantly associated with reduced OS [Figure 2(a)–(c)]. Interestingly, women had a better prognosis than men [Figure 2(d)], although they did not significantly differ regarding baseline characteristics as performance status, tumour stage and smoking status (data not shown).

Table 2 shows means of baseline lung function values in the total cohort as well in predefined survival cohorts (OS greater *versus* less or equal 2 years). With the exception of RV/TLC and DLCO%predicted the groups showed no significant differences. However, baseline values of the total cohort indicate that most patients might be affected by an impaired baseline lung function.

**Table 2.** Baseline lung function values. With exception of FEV_1_/FVC and RV/TLC all values represent percent of predicted value. All data are displayed as means ± standard deviation.

Variable	Total cohort (*N* = 205)	Survival > 2 years (*N* = 53)	Survival ⩽ 2 years (*N* = 152)	*p* Value
FEV_1_%predicted	66.9 ± 21.7	70.4 ± 22.3	65.6 ± 21.4	0.151
FVC%predicted	76.1 ± 21.2	78.9 ± 23.9	75.2 ± 20.2	0.342
FEV_1_/FVC (%)	68.9 ± 12.5	70.3 ± 11.1	68.4 ± 13.0	0.336
PEF%predicted	51.9 ± 17.7	60.7 ± 22.8	56.9 ± 22.8	0.293
sRtot%predicted	162.9 ± 123.9	167.4 ± 144.1	161.4 ± 117.1	0.660
RV%predicted	142.5 ± 49.2	130.5 ± 35.5	146.8 ± 52.8	0.126
TLC%predicted	97.0 ± 24.6	99.2 ± 25.5	96.2 ± 24.3	0.515
RV/TLC (%)	57.1 ± 19.5	51.1 ± 15.3	59.2 ± 20.4	**0.014**
DLCO%predicted	51.9 ± 17.7	56.6 ± 10.3	50.3 ± 19.3	**0.014**

Significant variables are printed bold. DLCO, diffusing capacity of the lungs for carbon monoxide; FEV_1_, forced expiratory volume in 1 s; FVC, forced vital capacity; PEF, peak expiratory flow; RV, residual volume; sRtot, specific resistance; TLC, total lung capacity.

### Airway obstruction does not predict survival in SCLC patients

**Figure 3.**
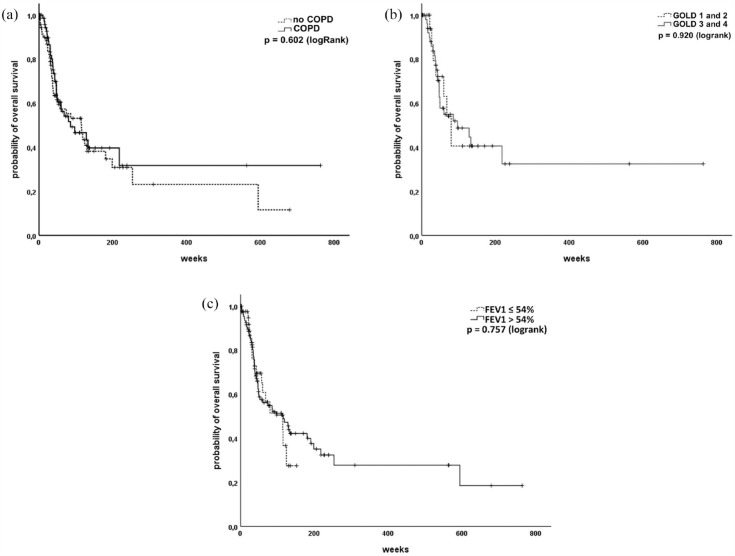
Bronchial obstruction does not predict survival in SCLC patients. (a) Comparison of patients with and without COPD. (b) COPD GOLD groups do not differ regarding their outcome in SCLC patients. (c) Patients with reduced FEV_1_ show no reduced outcome. COPD, chronic obstructive pulmonary disease; FEV_1_, forced expiratory volume in 1 s; SCLC, Small cell lung cancer.

In a first step, we sought to analyse whether airway obstruction at the time of SCLC diagnosis affects OS. We used different approaches to evaluate the effect of airway obstruction. Firstly, patients were grouped by the presence of the diagnosis of COPD according to GOLD recommendations defined as post-bronchodilator FEV_1_/FVC < 0.70.^18^ There was no difference in patients’ outcome depending on the diagnosis of COPD according to GOLD [Figure 3(a)]. Secondly, we compared patients of GOLD grades 1 and 2 with patients of higher GOLD grades 3 and 4 and found no differences in OS [Figure 3(b)]. In a third step, the ROC analysis revealed an FEV_1_ value of 54%predicted as optimal cut-off for the prediction of OS in our cohort. However, reduced FEV_1_ < 54% was also not predictive for poor survival [Figure 3(c)]. Other parameters of airway obstruction, in terms of reduced PEF or increased sRtot, were also not associated with reduced survival in our cohort (data not shown).

### Hyperinflation predicts higher mortality

Although the extent of pre-therapeutic airway obstruction did not correlate with patients’ outcome, we found that hyperinflation in terms of RV as well as RV/TLC was a strong prognostic factor. ROC analyses revealed 169.2% as an optimal cut-off for RV in our cohort. Therefore, we used 170% as cut-off for further analyses.^19^

Furthermore, severity of hyperinflation is often measured as relative hyperinflation, calculated by the RV/TLC quotient to correct for different lung volumes. The optimal cut-off for RV/TLC in our cohort evaluated by the ROC analysis was 59.2%. This is in line with previous investigations in COPD patients for the prediction of higher mortality using a cut-off for RV/TLC of 60% to define severe hyperinflation.^20,21^ Therefore, further analyses have been carried out using 60% as RV/TLC cut-off.

**Figure 4.**
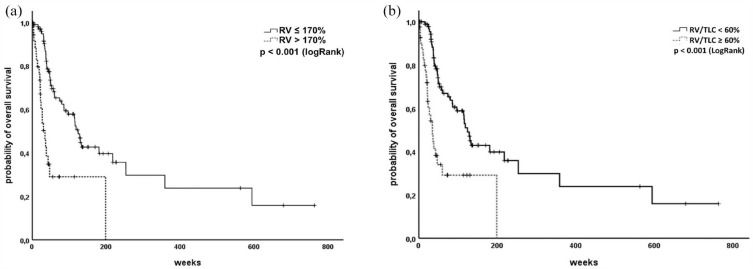
Hyperinflations predicts reduced overall survival. (a) Patients with increased RV have poorer outcome. (b) Relative hyperinflation significantly predicts survival in SCLC patients. RV, residual volume; SCLC, small cell lung cancer.

Patients with a residual volume of more than 170%predicted as well as increased RV/TLC ratio showed significantly reduced OS [Figure 4(a) and (b)]. As you can see in Figure 4, both definitions of hyperinflation comprise nearly the same patients leading to the same result.

### Reduced diffusing capacity is associated with shorter OS

**Figure 5.**
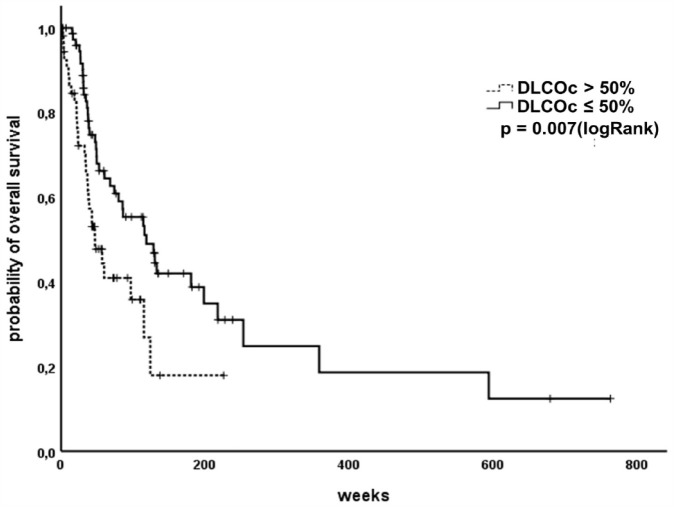
SCLC patients with reduced diffusing capacity show significantly reduced overall survival. SCLC, small cell lung cancer.

In a next step, we correlated impaired pre-therapeutic diffusing capacity (DLCOc) with survival in our cohort. ROC analyses predicted 51% as an optimal cut-off for OS in our cohort. Following previous recommendations, this meets the criteria of moderate impairment of DLCO (40–60%).^22^ It revealed that patients with reduced DLCOc (⩽50% of the expected value) showed significantly reduced OS (Figure 5).

### Impaired diffusing capacity and hyperinflation correlates with clinical emphysema phenotype

Our analyses revealed that patients with an emphysematic lung function pattern comprising hyperinflation and reduced diffusing capacity were affected by increased mortality. To evaluate whether these patients share a typical emphysematic clinical phenotype, we analysed different features of the emphysematic phenotype (low BMI, reduced performance status, smoking status). In our cohort, 28 patients were characterized by both RV/TLC ⩾ 60% and DLCO ⩽ 50% (emphysematic phenotype group), 36 patients harboured neither hyperinflation nor reduced diffusing capacity based on these definitions (non-emphysematic phenotype group), and 64 patients had one of both features (mixed group, Table 3). Patients allocated in the emphysematic phenotype group were significantly older compared to the non-emphysematic phenotype group (*p* = 0.007) or the mixed group (*p* < 0.001). Furthermore, the emphysematic phenotype group showed significantly lower BMI compared to non-emphysematic patients (*p* = 0.031), whereas there was no significant difference between the emphysema group and the mixed group. Additionally, ECOG-Performance status was significantly worse in patients of the emphysema group compared to non-emphysematic phenotype group (*p* = 0.015) and mixed (*p* = 0.001). No significant difference regarding smoking status or tumour stage was found between the three groups.

**Table 3.** Characteristics and comparison of patients classified regarding their emphysematic phenotype.

Variable	Emphysema	Non-emphysema	Emphysema *versus* Non-emphysema	Mixed	Emphysema *versus* mixed
	*n* = 28	*n* = 36		*n* = 64	
Gender					
Male	16 (57.1)	19 (52.8)		40 (62..57)	
Female	12 (42.9)	17 (47.2)		24 (37.5.)	
Age	71.1 ± 8.4	64.8 ± 8.1	***p*** **=** **0.007**	63.4 ± 9.0	***p*** **<** 0.001
BMI	24.1 ± 5.2	25.7 ± 4.2	***p*** **=** **0.053**	25.2 ± 4.0	*p* = **0.030**
Initial Eastern Cooperative Oncology Group Performance Status (ECOG)					
0	1 (3.6)	10 (27.8)	***p* = 0.015**	17 (26.6)	***p* = 0.001**
1	7 (25.0)	14 (38.9)		29 (45.3)	
2	10 (35.7)	5 (13.9)		5 (7.8)	
3	3 (10.7)	1 (2.8)		0 (0)	
4	1 (3.6)	1 (2.8)		0 (0)	
Unknown	6 (21.4)	15 (21.7)		13 (20.3)	
Smoking status					
Former smoker	9 (32.1)	15 (41.7)	*p* = 0.477	19 (29.7.7)	*p* = 1.0
Current smoker	13 (46.4)	14 (38.9)		28 (43.8)	
Never smoker	1 (3.6)	(0)		1 (1.6)	
Unknown	5 (17.9)	7 (19.4)		16 (25.0)	
UICC Stage					
I	1 (3.6)	0 (0)	*p* = 0.509	0 (0)	*p* = 0.122
II	1 (3.6)	2 (5.6)		8 (12.5)	
III	4 (14.3)	9 (25.0)		15 (23.4)	
IV	22 (78.6)	25 (69.4)		39 (60.9)	
Unknown	0 (0)	0 (0)		2 (3.1)	
Cardiovascular disease					
Yes	15 (53.6)	17 (47.2)	*p* = 0.950	32 (50)	*p* = 0.753
No	13 (4.,4)	19 (52.8)		32 (50)	

Significant variables are printed bold.

### Impaired diffusing capacity and hyperinflation significantly predict poor OS

**Figure 6.**
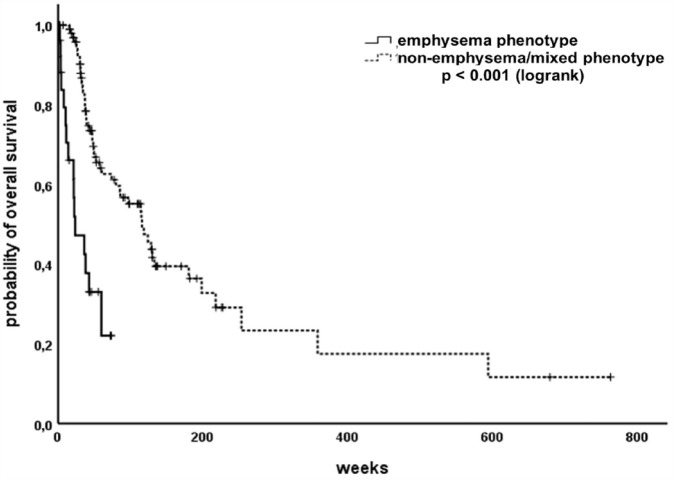
Patients with emphysematic phenotype show significantly reduced overall survival.

Combining RV/TLC ⩾ 60% and DLCO ⩽ 50%predicted, to define an emphysematic phenotype, resulted in a highly significant association with survival in our SCLC cohort. Patients classified into the emphysema group showed significantly reduced OS compared to the both other groups (*p* < 0.001, Figure 6).

We performed COX-regression to test both variables RV/TLC and DLCO in a multivariate model. It revealed that RV/TLC is a highly significant independent variable [HR 5.88; 95% CI (2.18–15.85); *p* < 0.001], whereas DLCO was found to be not significant in the multivariate model [HR 0.99; 95% CI (0.98–1.01); *p* = 0.407].

## Discussion

In this retrospective study, we analysed a cohort of 205 patients diagnosed with SCLC between 2010 and 2018. With the majority of SCLC patients being smokers, which is on the other hand also closely linked to the comorbidity COPD, we sought to examine whether we could find prognostic markers for OS in pre-therapeutic lung function testing.

Our study population is a classic SCLC cohort being predominantly male, predominately smokers and 90% of patients diagnosed in advanced tumour stages. This is in line with previous investigations on SCLC patients.^23^

This study indicates that the presence of airway obstruction based on spirometric lung function testing using FEV_1_ and FEV_1_/FVC predicted as well as the different GOLD stages 1–4 did not affect outcome in our SCLC cohort. This is in line with previous publications, that is, Ju *et al.* who analysed 110 SCLC patients receiving chemotherapy.^24^ In this trial, the diagnosis of COPD had no impact on patients’ survival.^24^ The same results were found in a recent Korean study.^13^ A large study evaluated the impact of COPD in 9425 SCLC patients and found a slight survival advantage for patients without COPD in the first year. However, this trend vanished after a follow-up of 1 year.^25^ Another study with 449 lung cancer patients found no prognostic impact of COPD in either NSCLC or SCLC.^11^ However, in all these publications, the diagnosis COPD is only based on medical history or spirometric lung function data. The diagnosis of COPD is clearly linked to the development of SCLC,^8,26,27^ but until now there is no strong evidence, that the comorbidity COPD affects prognosis for SCLC patients.

Although NSCLC patients with COPD (FEV_1_/FVC < 0.7) and a FEV_1_ < 80%predicted had a poorer outcome, there was no comparable effect in SCLC.^11^ Kang *et al.* evaluated 170 SCLC patients with pre-therapeutic lung function testing and found no prognostic impact of COPD in their cohort. However, the study showed an association of FEV_1_ < 80%predicted with slightly decreased survival.^10^ In our study, FEV_1_ < 54%predicted was the optimal cut-off value for the prediction of survival based on ROC analyses, but the comparison of patients with FEV_1_ < 54%predicted *versus* ⩾54%predicted showed no significant difference regarding patients’ survival.

We found only one investigation, which indicated a prognostic impact of reduced FEV_1_/FVC ratio in limited disease SCLC.^9^ This might be due to the fact that this cohort of limited disease patients consists of more long-term survivors. Furthermore, many of these patients received thoracic irradiation and impaired lung function increases the risk for radiation induced pulmonary complications.^28^

Therefore, our results are in line with previous data that airway obstruction and COPD does not generally affect prognosis in SCLC patients, and spirometric lung function values are not predictive for SCLC survival.

On the other hand, our analysis adds to the existing knowledge that hyperinflation and reduced diffusing capacity is strongly correlated with poor prognosis in SCLC patients. Patients with both conditions, representing an emphysematic phenotype, had the worst outcome.

Analysing optimal cut-offs by ROC analysis, we could confirm existing cut-offs defining sever hyperinflation and moderate impairment of diffusing capacity. For example, the cut-off for severe hyperinflation following the guideline of the German society of pneumology (DGP) defining severe hyperinflations as RV greater than 170% fits with our analysis. This strengthens our results because recommended evaluation of lung function testing correlates with the outcome of SCLC patients.

To our knowledge, there are no previous publications investigating the prognostic impact of extended pre-therapeutic lung function testing including body plethysmography and measurement of diffusing capacity in SCLC patients. Until now, these procedures are internationally not standard of care in pre-therapeutic assessment of SCLC patients. There are few data, that lung emphysema predisposes for the development of lung cancer,^26,29^ but the consequences of this condition for the prognosis of lung cancer patients have not been evaluated yet.

Based on our study data, a relevant gain in information is shown by the additional implementation of body plethysmography and diffusing capacity for the evaluation of the overall SCLC. The identification of patients with severe hyperinflation and moderate impairments in gas exchange my indetify patients at risk of early deterioration. This information should be taken into account not only for a more personalized approach in treating the cancer but also the patient from a multidisciplinary perspective. Therefore, the comorbid condition of lung emphysema also needs consequent respiratory physiotherapy and optimal bronchodilator treatment to reduce hyperinflation, as well as nutritional counselling in particular in patients with pre-existing cachexia. With former data indicating that smoking cessation, especially in limited diseases SCLC leads to improved survival,^30^ we also recommend strict efforts for smoking cessation in these patients.

The study is subject to the following limitations. First of all, the retrospective character of the analysis might lead to introduce a selection bias. Furthermore, our cohort comprised SCLC patients initially diagnosed with SCLC between 2010 and 2018. The integration of checkpoint-inhibitors and modern irradiation procedures have changed the treatment of SCLC patients in the last years. However, this is the first study reporting a clear correlation of extended lung function testing and survival in SCLC patients. These results should be validated in a prospective cohort.

## Conclusion

This study evaluated the impact of extended pre-therapeutic lung function testing in unselected SCLC patients. Airway obstruction measured by spirometric lung function data did not affect survival in this cohort. However, an emphysematic phenotype in terms of hyperinflation and impaired gas exchange was associated with less OS in SCLC patients. The most informative cut-off values were RV/TLC ⩾ 60% as well as DLCOc ⩽ 50%. Therefore, we recommend extended pre-therapeutic assessment of SCLC patient by body plethysmography and measurement of diffusing capacity. Furthermore, consequent respiratory physiotherapy, optimization of bronchodilator treatment and smoking cessation might reduce hyperinflation in these patients and may lead to better outcomes.

## Declarations

**Ethics approval and consent to participate:** The project was approved by the ethical committee of the University of Munich (LMU). As this is a retrospective study, in which all data have been anonymized after obtainment, no patient consent was necessary.

**Consent for publication:** Not applicable.

**Availability of data and materials:** Not applicable.
